# Protocol: a fast, comprehensive and reproducible one-step extraction method for the rapid preparation of polar and semi-polar metabolites, lipids, proteins, starch and cell wall polymers from a single sample

**DOI:** 10.1186/s13007-016-0146-2

**Published:** 2016-11-10

**Authors:** Mohamed A. Salem, Jessica Jüppner, Krzysztof Bajdzienko, Patrick Giavalisco

**Affiliations:** 1 Max Planck Institute of Molecular Plant Physiology, Am Mühlenberg 1, 14476 Potsdam-Golm, Germany; 2 Department of Pharmacognosy, Faculty of Pharmacy, Cairo University, Kasr El-Aini Street, Cairo, 11562 Egypt

**Keywords:** Primary metabolites, Secondary metabolites, Lipids, MTBE, Starch, Cell wall, Proteins, Metabolomics, Lipidomics, Proteomics, Systems biology

## Abstract

**Background:**

The elucidation of complex biological systems requires integration of multiple molecular parameters. Accordingly, high throughput methods like transcriptomics, proteomics, metabolomics and lipidomics have emerged to provide the tools for successful system-wide investigations. Unfortunately, optimized analysis of different compounds requires specific extraction procedures in combination with specific analytical instrumentation. However, the most efficient extraction protocols often only cover a restricted number of compounds due to the different physico-chemical properties of these biological compounds. Consequently, comprehensive analysis of several molecular components like polar primary metabolites next to lipids or proteins require multiple aliquots to enable the specific extraction procedures required to cover these diverse compound classes. This multi-parallel sample handling of different sample aliquots is therefore not only more sample intensive, it also requires more time and effort to obtain the required extracts.

**Results:**

To circumvent large sample amounts, distributed into several aliquots for the comprehensive extraction of most relevant biological compounds, we developed a simple, robust and reproducible two-phase liquid–liquid extraction protocol. This one-step extraction protocol allows for the analysis of polar-, semi-polar and hydrophobic metabolites, next to insoluble or precipitated compounds, including proteins, starch and plant cell wall components, from a single sample. The method is scalable regarding the used sample amounts but also the employed volumes and can be performed in microcentrifuge tubes, enabling high throughput analysis. The obtained fractions are fully compatible with common analytical methods, including spectroscopic, chromatographic and mass spectrometry-based techniques. To document the utility of the described protocol, we used 25 mg of *Arabidopsis thaliana* rosette leaves for the generation of multi-omics data sets, covering lipidomics, metabolomics and proteomics. The obtained data allowed us to measure and annotate more than 200 lipid compounds, 100 primary metabolites, 50 secondary metabolites and 2000 proteins.

**Conclusions:**

The described extraction protocol provides a simple and straightforward method for the efficient extraction of lipids, metabolites and proteins from minute amounts of a single sample, enabling the targeted but also untargeted high-throughput analyses of diverse biological tissues and samples.

**Electronic supplementary material:**

The online version of this article (doi:10.1186/s13007-016-0146-2) contains supplementary material, which is available to authorized users.

## Background

Systems biology, the comprehensive study of several biological components and the analysis of their complex dependencies within a biological cell or tissue [1], is an indispensable approach to understand complex cellular functions and processes. To obtain the analytical data for the diverse molecular constituents, ‘-omic’ platforms, including transcriptomics [2], metabolomics [3, 4], lipidomics [5, 6] and proteomics [7, 8] have emerged to provide the ever growing tool-box for successful systems biology investigations [9]. Metabolomics and lipidomics are aiming for the identification and quantification of the complement of all small molecules and lipids within a biological system, respectively [10]. In recent years, metabolomics and lipidomics have emerged as cornerstones in the field of systems biology [11].

Owing, not only to the complexity, but also to the diverse physico-chemical properties of the cellular constituents, especially the different metabolite classes, no single extraction solvent can extract all molecular components from a complex biological sample [12, 13]. Accordingly, different classes of compounds require specific extraction methods to obtain adequate coverage of the full diversity of cellular metabolism [14, 15].

Because of the above-mentioned extraction specificity, multiple aliquots of the same sample are required to obtain sufficient material for the different extraction procedures. Next to the increased effort due to multi-parallel sample handling, the required sample amounts for multiple extractions are often not available for all tissues or organisms. Consequently, a comprehensive extraction method providing the robust and reliable recovery of the major molecular components from a single aliquot of a single sample would be desirable. Such a method would decrease the sample handling time and therefore increase the sample throughput. Since the compounds are derived from the same aliquot, it would also improve the data precision and its comparability.

To minimize the problem of multiple extractions from several sample aliquots, multi-phase extraction protocols, often relying on a two-phase separation system, consisting of different mixtures of chloroform and methanol, have been developed. These methods were initially designed for the extraction and analysis of either pure lipids [16, 17] or polar metabolites [18]. Accordingly, the motivation to use the two-phase separation methods of these initial studies was to reduce the compound complexity in the extracted sample and clean them up from compounds possibly negatively interfering with downstream analytical methods, leading to improved quality in the analysis of the compounds of interest. Only later, with the onset of system-wide analysis strategies, these methods, especially the chloroform–methanol extraction protocol, were also projected to collect more than a single fraction of the two main phases [18–21]. Unfortunately, the main problem associated with the reproducibility of the chloroform-based methods is derived from the fact that the solid components lie between the upper organic (chloroform) and the lower methanol/water phase after the phase separation. Accordingly, this diffuse and amorphous interphase hinders the quantitative collection of this insoluble fraction, but also complicates the maximal and contamination-free collection of the two main liquid phases. To overcome this problem, recent modifications of the chloroform–methanol method were introduced. Here the phase separation of the homogeneous, one phase chloroform–methanol mixture, was achieved only after a centrifugation and the separation of the solid and the liquid phase in an independent step [18–21]. This two-step approach partially overcomes the problem of the interphase between the two liquid phases but it is more time consuming, since the phase separation has to be achieved in an independent step [22]. Additionally, unwanted phase separation could occur if the water content of the extracted samples are too high.

To overcome these problems, we used a cleaner and safer alternative to chloroform, namely methyl *tert*-butyl ether (MTBE) for liquid–liquid extraction [23]. MTBE was initially used for the recovery of bacterial organic acids [24] and lipids from different eukaryotic samples [25, 26]. The main advantage of using MTBE is the fact that it has a severely decreased density (0.74 g cm^−3^) compared to chloroform (1.48 g cm^−3^), which not only leads to an inversion of the methanol and the MTBE phases, but also to a stable and solid pellet at the bottom of the centrifugation tube. Based on these improved separation features, we were able to set up a MTBE-based extraction method for the complete recovery of multiple compounds [23].

In this article, we now summarize and describe the complete single-step extraction protocol for rapid comprehensive and simultaneous analysis of lipids, metabolites and proteins from a single aliquot of plant tissues. This protocol also includes a reproducible recovery of starch and cell wall (CW) polymers from the solid phase. Using *Arabidopsis thaliana* leaves as a model, we generated lipidomics, metabolomics and proteomics datasets from 25 mg sample. We have successfully used this method to annotate more than 200 lipid compounds, covering most of the classes involved in lipid metabolism. Additionally, we annotated more than 50 compounds using LC–MS method covering most of phenylpropanoids and glucosinolates and more than 90 covering the classes involved in central metabolism from GC–MS method. Additionally, we obtained about 2000 protein identifications but also the polysaccharide composition of the cell wall and the crystalline cellulose content. We therefore believe that this method could be used, with minor adaptations, to analyze metabolites, lipids and proteins from most biological samples.

## Methods

### Plant material

*Arabidopsis thaliana* seeds (wild-type of ecotype Col-0) were stratified at 4 °C in dark for 3 days before sowing them on soil. The plants were grown in long day (LD) phytotrons that were maintained at 16/8 light/dark cycle. The average light intensity was maintained at 150 µmol m^−2^/s^2^. The day/night temperature and relative humidity were 20/16 °C and 60/75%, respectively. Rosette leaves of 21-day-old plants were harvested and snap frozen in liquid nitrogen. The plant material was grounded into a homogeneous and fine powder using tissue homogenizer and then aliquoted (25 mg) into 2 ml safe-lock microcentrifuge tubes.

### Reagent set-up

For the preparation of 100 ml of extraction solvent mixture 1 (M1), 75 ml of methyl *tert*-butyl ether were added to 25 ml of methanol (3:1, vol/vol). Corticosterone (50 µl of a 1 mg/ml stock solution in methanol) and ampicillin (25 µl of a 1 mg/ml stock solution in methanol) were used as internal standards for UPLC-MS analysis of semi-polar metabolites. 1,2-diheptadecanoyl-*sn*-glycero-3-phosphocholine (50 µl of a 1 mg/ml stock solution in chloroform) and ^13^C sorbitol (50 µl of a 1 mg/ml stock solution in water) were added as internal standards for the UPLC-MS analysis of lipid and GC–MS analysis of polar metabolites, respectively. For extraction solvent mixture 2 (M2), phase separation-inducing solvent, 75 ml of water were added to 25 ml of methanol (3:1, vol/vol).

### Extraction and phase separation

A fixed volume (1 ml) of pre-cooled (−20 °C) extraction solvent M1 was added to homogenized tissues. After adding the extraction solvent, the vials/tubes were thoroughly vortexed for 1 min and then incubated on an orbital shaker (100 rpm) for 45 min at 4 °C followed by a 15 min sonication step. For phase separation, a volume of 650 µl of solvent M2, was added to each vial/tube and the samples were again thoroughly vortexed for 1 min. After that, the samples are centrifuged at a speed of 20,000*g* for 5 min at 4 °C.

### Analysis of lipids from the MTBE-phase by UPLC-MS

A fixed volume (500 µl) of the solvent from the upper, lipid-containing phase, was transferred to a pre-labelled 1.5 ml microcentrifuge tube or glass vial and the solvent was evaporated using either a SpeedVac concentrator at RT or, preferably, a nitrogen flow evaporator. For the lipidomic analysis, we used our previously published Ultra Performance Liquid Chromatography Mass Spectrometry (UPLC-MS) method [27]. Briefly, the dried pellets from the 500 µl lipid fractions were re-suspended in 250 µl acetonitrile: 2-propanol (7:3, vol/vol) solution. Once the samples are re-suspended in appropriate volumes, 2 µl per sample was injected and the lipids were separated on a Reversed Phase (RP) Bridged Ethyl Hybrid (BEH) C_8_ column (100 mm × 2.1 mm containing 1.7 μm diameter particles, Waters), using a Waters Acquity UPLC system (Waters, Machester, UK). The mass spectra were acquired in positive and negative ionization mode using a heated electrospray ionization (HESI) source in combination with an Exactive, Orbitrap-type, MS (Exactive, Thermo-Fisher, Bremen, Germany). The mobile phases used for the chromatographic separation were water containing 1% 1 M ammonium acetate, 0.1% acetic acid (Buffer A) and acetonitrile: isopropanol (7:3, vol/vol) containing 1% 1 M ammonium acetate, 0.1% acetic acid (Buffer B). The gradient separation was: 1 min 45% A, 3 min linear gradient from 45% A to 35% A, 8 min linear gradient from 25% A to 11% A, and 3 min linear gradient from 11% A to 1% A. After washing the column for 3 min with 1% A, the buffer is set back to 45% A and the column is re-equilibrated for 4 min. The flow rate was set to 400 µl/min. Data analysis was performed using the Progenesis QI software package (Progenesis QI Version 2.2, Nonlinear Dynamics, Newcastle, UK) and as described in Hummel et al. [27].

### Analysis of pigments from the MTBE-phase

To measure chlorophylls, a volume of 100 µl of the upper MTBE phase was mixed with 900 µl of methanol (1:9, vol/vol). The absorption UV–VIS spectra were measured and the concentration of chlorophyll a (Chl_a_), chlorophyll b (Chl_b_), total chlorophylls (Chl_a+b_), and total carotenoid contents was calculated as described previously [28–30]. Additionally, a volume of 200 µl of the upper MTBE phase was evaporated and used for HPLC-based analysis of carotenoids [31].

### Analysis of primary metabolites from the methanol/water phase by GC–MS

After having removed the remaining lipid phase from the vials/tubes, 200 µl of the polar phase was transferred into pre-labelled 1.5 ml microcentrifuge tube and the samples were dried down in a SpeedVac concentrator without heating. For the analysis of the samples, the dried pellets were derivatized and analyzed using a previously published GC-TOF–MS method [21, 32]. Briefly, the dried 200 µl aliquots of the polar phase were re-suspended in methoxyamine-hydrochloride/pyridine solution for methoxymization of carbonyl groups followed by heating at 37 °C for 90 min. The samples were further derivatized with *N*-methyl-*N*-trimethylsilyltrifloracetamide (MSTFA) for 30 min at 37 °C. The MSTFA solution contained a mixture of 13 fatty acid methyl esters (FAMEs) with different chain length, which were used in the post-measurement as retention time standards. 1 µl of the derivatized sample mixture was injected onto the GC-column and measured. Data analysis was performed using the TargetSearch package according to Cuadros-Inostroza et al. [33].

### Analysis of secondary metabolites from the methanol/water phase by UPLC–MS

A fixed volume of 400 µl of the polar phase was transferred into a pre-labelled 1.5 ml microcentrifuge tube and the samples were dried down in a SpeedVac concentrator without heating. For the direct analysis, the samples were handled as described previously in Giavalisco et al. [23]. Briefly, the dried 400 µl aliquots of the polar phase were re-suspended in 200 µl UPLC-grade methanol: water (1:1, vol/vol) and transferred to the autosampler, 2 µl was injected and separated on RP High Strength Silica (HSS) T3 C_18_ column (100 mm × 2.1 mm containing 1.7 μm diameter particles, Waters), using a Waters Acquity UPLC system. The mass spectra were acquired by full scan MS in positive and negative ionization mode on an Exactive high resolution Orbitrap-type MS (Thermo-Fisher, Bremen, Germany). The mobile phases used for chromatographic separation were water containing 0.1% formic acid (Buffer A) and acetonitrile containing 0.1% formic acid (Buffer B). The compounds were separated by a gradient: 1 min 99% A, 13 min linear gradient from 99% A to 65% A, 14.5 min linear gradient from 65% A to 30% A, 15.5 min linear gradient from 30% A to 1% A, hold 1% A until 17, 17.5 min linear gradient from1% A to 99% A, and re-equilibrate the column for 2.5 min. The flow rate was adjusted to 400 µl/min. Data analysis was performed by using the Progenesis QI software package (Progenesis QI Version 2.2, Nonlinear Dynamics, Newcastle, UK).

### The sequential extraction of protein and starch from the insoluble pellet

For the sequential protein and starch extraction, the remainder of the aqueous phase was removed by pipetting off the excess volume. The obtained pellet after the metabolite and lipid extraction was washed by thoroughly adding 500 µl methanol and vortexing the samples for 30 s. The samples were centrifuged at a speed of 20,000*g* for 5 min at 4 °C. This washing step was repeated two more times.

### Extraction and analysis of proteins by LC–MS/MS

For protein extraction, the washed pellet of a 25 mg leaf material was re-suspended in 150 µl of protein extraction buffer (6 M urea, 2 M thiourea, 15 mM DTT, 2% CHAPS and protease and phosphatase inhibitors). Once the proteins were dissolved, the samples were sonicated for 10 min in a sonication bath, followed by an additional 30 min incubation on an orbital shaker (100 r.p.m.) at room temperature. In the next step, the solubilized proteins were centrifuged at 10,000*g* for 5 min and the protein concentration was determined from the collected supernatant [34]. 50 µg of proteins extract were digested in-solution using a Trypsin/Lys-C mixture (Mass Spec Grade, Promega) according to the instruction manual. After the digestion, the samples were desalted using C_18_ stage tips as described in Rappsilber et al. [35]. After the elution of the digested and desalted peptides from C_18_-stage tips, the samples were concentrated to near dryness in a SpeedVac and the peptide mixtures were analyzed by LC-MS/MS using a Q ExactivePlus high resolution mass spectrometer connected to an EASY-nLC 1000 system (Thermo-Fisher, Bremen, Germany). Peptides were separated using a binary buffer system of 0.1% formic acid in water (Buffer A) and 60% acetonitrile containing 0.1% formic (Buffer B). The flow rate was adjusted to 300 nl/min. Peptides were eluted with using a linear gradient of 0–40% buffer B for 50 min followed by a linear gradient between 40–80% buffer B for additional 30 min. Peptides were analyzed with one full scan (200–2000 *m*/*z*, R = 70,000 at 200 *m*/*z*), followed by up to fifteen data-dependent MS/MS scans (Top 15 approach) with higher-energy collisional dissociation (HCD) at a resolution of 17,500 at 200 *m*/*z*. Dynamic exclusion was set to 30 s. Raw data were processed using the Progenesis QI for proteomics (Progenesis QI for Proteomics Version 3.0, Nonlinear Dynamics, Newcastle, UK) software in combination with the Mascot (Version 2.5, MatrixScience, Boston MS, USA) database search tool using the Arabidopsis TAIR database (Version 10, The Arabidopsis Information Resource, www.arabidopsis.org).

### Extraction and enzymatic determination of the starch content

For starch extraction, the remaining pellet after protein extraction was washed using 1 ml of 80% ethanol. After that step, the samples were incubated for 3 min at 80 °C and finally centrifuged at 3000*g* for 10 min at room temperature. The washed pellets were re-dissolved in 0.5 ml of water and the starch was gelatinized by heating at 100 °C for 1.5 h. After allowing the samples to cool, 0.5 ml of 200 mM sodium acetate was added and the dissolved starch was digested into its glucose monomers with an enzyme mix of α-amyloglucosidase and α-amylase, according to manufacturer instructions [36, 37]. The tubes were incubated overnight at 37 °C and finally centrifuged at 10,000*g* for 5 min at room temperature. Glucose concentration was determined based on an enzymatic assay through hexokinase and glucose 6-phosphate dehydrogenase and the assay was performed in a 96-well plate using a microtiter plate reader. Briefly, an appropriate volume (40 µl) of the digested samples was mixed with 160 µl of glucose assay mix consists of 100 mM HEPES, pH 7.5, 4 mM MgCl_2_, 0.5 mM adenosine triphosphate (ATP) and 1 mM nicotinamide adenine dinucleotide (NAD^+^), hexokinase (6 U/ml). After monitoring the initial absorption at 340 nm (OD_340_), 0.25 units glucose 6-phosphate dehydrogenase were added to each well and the OD_340_ was recorded again. Starch concentration was determined based on a calibration curve of a standard glucose [36, 37].

### Analysis of cell wall composition

For the analysis of the cell wall polymers, the remaining pellet, after protein and starch extraction, was washed three times by thoroughly vortexing the samples for 30 s in 500 µl of water. After washing the pellets, the samples were air-dried in a container with silica beads and were analyzed immediately or they can be stored in a desiccator until further extraction.

The detailed polysaccharide composition of cell walls was determined after acid hydrolysis and GC derivatization [38, 39]. Briefly, 2 mg of the cell wall pellet was hydrolyzed by dissolving in 200 µl of 2.5 M trifluoroacetic acid (TFA) and heating at 121 °C for 1.5 h. The samples were allowed to cool before centrifuging them at 10,000*g* for 5 min at room temperature. An appropriate volume (100 µl) of the acidic supernatant was transferred to new glass screw-capped tubes and 10 µl of the internal standard (10 mg/ml of *myo*-inositol) was added. Samples were evaporated to dryness, reduced, acetylated and finally measured on GC–MS [38, 39]. The content of crystalline cellulose was determined by a spectrophotometric method [39, 40]. Briefly, the pellet remained after hydrolysis with TFA was re-dissolved in 100 µl of acetic acid/nitric acid/water (8: 1: 2, vol/vol/vol). The samples were quickly vortexed, heated at 100 °C for 30 min, cooled to room temperature and finally centrifuged at 10,000*g* for 10 min at room temperature. After discarding the supernatant, the pellet was washed tree times with 100 µl of water and finally re-dissolved in 100 µl of 72% sulfuric acid. Crystalline cellulose content was determined based on glucose standard curve using the colorimetric anthrone assay [39, 40].

The lignin content and composition was determined using the thioglycolic acid (TGA) and the thioacidolysis quantification methods, respectively [41–44]. For TGA quantification of lignin, 1 mg of the prepared cell wall material was re-suspended in 250 µl of 2 N HCl and 25 µl of TGA and the samples were incubated at 100 °C for 3 h with regular shaking. The samples were allowed to cool before centrifuging them at 10,000*g* for 5 min at room temperature. The pellet was washed three times with 0.5 ml of water before re-dissolving in 0.5 ml of 1 M NaOH followed by overnight incubation at room temperature with gentle shaking. The sample were centrifuged at 12,000*g* for 10 min at room temperature and the supernatant was acidified with 100 µl of concentrated HCl before incubating them at 4 °C for 4 h with regular shaking. The pellet remained after centrifugation was re-dissolved in 1 ml of 1 M NaOH and then the absorbance was measured spectrophotometrically at 280 nm [41, 42]. For lignin composition, 1 mg of the prepared cell wall material was re-suspended in 100 µl of 2.5% boron trifluoride etherate and 10% ethanethiol/dioxane solution. The samples were heated at 100 °C for 4 h with shaking. The samples were allowed to cool before adding 100 µl of 0.4 M sodium bicarbonate followed by liquid–liquid separation using 0.5 ml of ethyl acetate and 1 ml of water. An appropriate aliquot (200 µl) of the ethyl acetate layer was allowed to evaporate followed by derivatization and GC–MS analysis [43, 44].

### Troubleshooting

During the development and validation of this protocol, a number of issues arose, for which we developed a troubleshooting guide, which is summarized in Additional file 1: Table S1.

## Results

### Development of a comprehensive extraction method

**Fig. 1:**
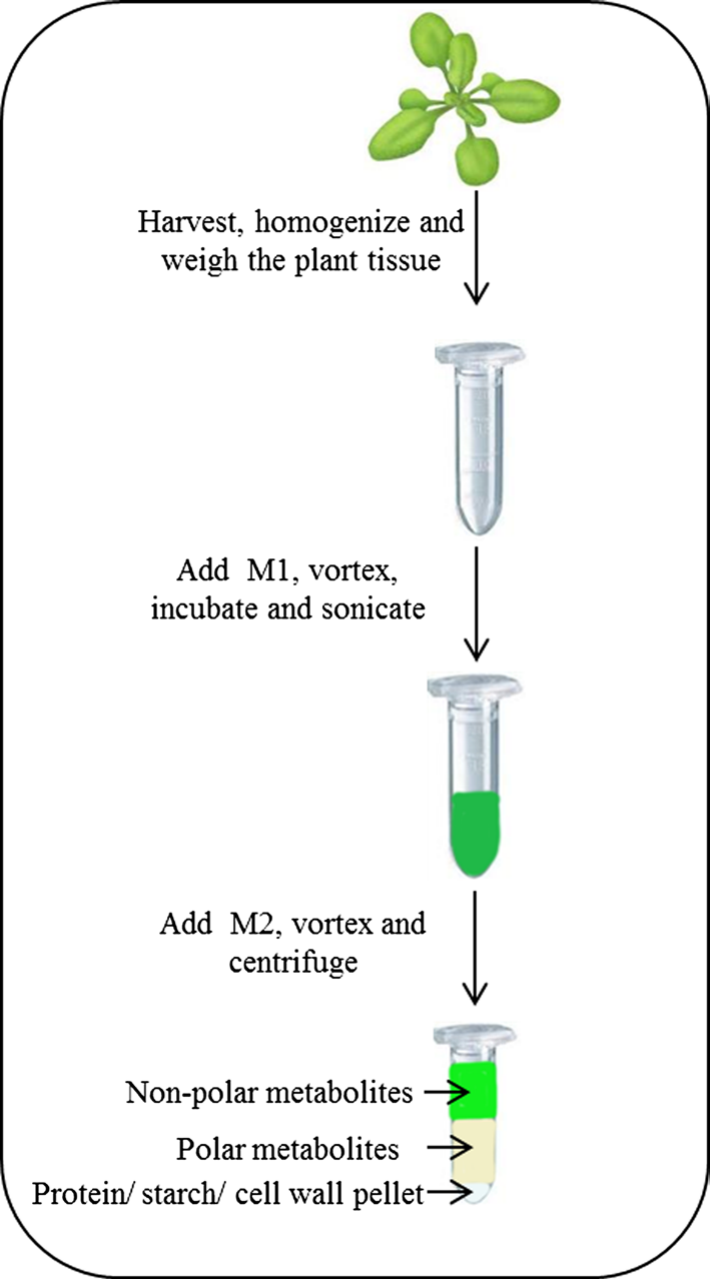
Overview of the experimental workflow for the MTBE-based extraction procedure. Plant material is harvested and snap-frozen in liquid nitrogen. The harvested tissue is homogenized using a pre-cooled mortar and pestle or cooled tubes in a mixer mill. About 10–50 mg ±10% of the frozen powder is weighed in pre-labelled 2 ml microcentrifuge tube. The weighed powder is extracted using 1 ml of the first extraction solvent (M1, MTBE:MeOH 3:1, vol/vol) followed by rigorous vortexing, agitated incubation and sonication of the samples. A liquid/liquid phase separation is achieved by adding 0.65 ml of the second extraction solvent (M2, H2O:MeOH 3:1, vol/vol) followed by vortexing and centrifugation

Based on the requirement to extract all relevant molecular features from a biological sample, ideally from a single sample aliquot, we decided to develop a comprehensive one-step extraction protocol for the analysis of plant tissue. The developed liquid–liquid two-phase separation system, which is conceptual similar to the classical chloroform: methanol extraction methods [18–21], relies on a MTBE: methanol: water system. Based on the initially published version of the method, where we analyzed lipids, proteins and polar metabolites [23], we were able to extend and improved our previously published extraction protocol. The updated protocol allows for the fast and reproducible extraction of lipids, pigments, polar to semi-polar primary and secondary metabolites but also proteins, starch and cell wall (CW) polymers. Figure 1 illustrates graphically the simple and straightforward workflow of the described extraction protocol, which is easily adjustable to the required amount of sample. Usually between 10 and 50 mg of tissue are used. The employed sample amounts are depending on tissue availability but also the intended analysis. Tissue amounts within this scale are routinely extracted in 2 ml microcentrifuge tubes, using 1 ml of extraction buffer M1 and 0.65 ml of extraction buffer M2 (see “Methods” section). If larger amounts of tissue have to be extracted, the extraction volume can be linearly scaled. Unfortunately, larger extraction volumes cannot be handled any longer in microcentrifuge tubes, which decreases the throughput of the method. Thus far, we have not encountered biological material where the usage of larger amounts of material for the comprehensive analysis of lipids, polar metabolites or secondary metabolites was required, actually contrary we were able to extract a full lipid profile from as little as 20 *Arabidopsis thaliana* seeds (data not shown).

**Fig. 2:**
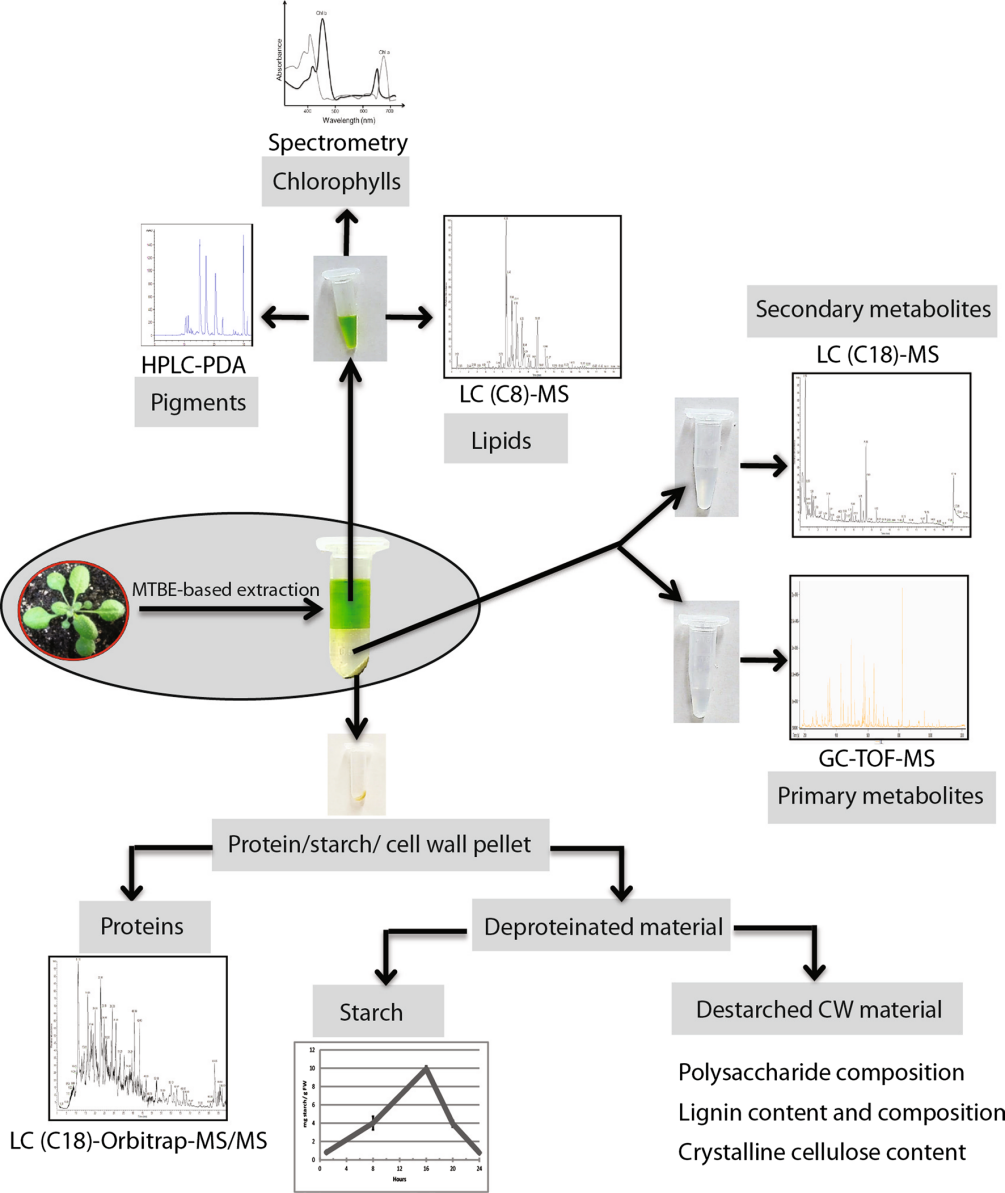
Schematic overview of the applied analytical methods. Following the two plus one phase extraction procedure, a phase separation of upper non-polar metabolites and a lower layer of polar to semi-polar metabolites next to a solid pellet (proteins, starch and cell wall) is obtained. A predefined volume (0.5 ml) of the upper lipid phase is aliquoted into three fractions (0.2, 0.2 and 0.1 ml), which are concentrated and analyzed by UPLC-MS, LC- photodiode array (PDA) or spectrometry for the lipid, pigment or chlorophyll composition, respectively. Two aliquots with predefined volume (0.2 and 0.4 ml) of the lower methanol: water phase are dried and the re-suspended compounds are analysed by GC- and UPLC-MS for analysis of primary and secondary metabolite composition, respectively. The starch/protein/cell wall pellet is washed followed by sequential protein and starch extraction. The de-proteinated and de-starched pellet, which contains the remaining cell wall material, can be used for determination of polysaccharide composition, cellulose and lignin

As indicated in Fig. 1, the whole procedure requires only few pipetting steps once the required sample are aliquoted in the microcentrifuge tubes. Due to this simplified workflow, a single person can handle 100 or more samples within half working day (4 h), enabling high throughput sample preparation as a pre-requirement for large-scale experiments. In the following sections we provide an exemplary multi-omics analysis of a 25 mg leaf sample of *Arabidopsis thaliana* (Col-0), extracted with 1 ml of the MTBE: Methanol extraction solution. Figure 2 provides an overview of the analytical workflow applied to the different fractions of the tissue sample.

### Analysis of the lipid phase

As indicated in the Fig. 2, the upper-organic (MTBE) phase obtained from the extraction contains most of the hydrophobic compounds. On the one hand, as indicated by the green color of the extract, this fraction contains the full inventory of pigments, including the major chlorophylls, but also several carotenoids. On the other hand, most lipids, namely the polar phospholipids and sphingolipids, which are the constituents of the cellular membrane system, next to the neutral storage lipids and the free fatty acids were extracted in this phase.

To validate this hydrophobic phase of the MTBE-extraction protocol, we initially tested the efficiency but also the reproducibility of this fraction compared to other commonly used protocols used for specific analysis of hydrophobic metabolites. For this purpose, we have performed parallel extractions of chlorophylls using the organic MTBE-fraction and compared the obtained results to chlorophyll extraction methods using acetone [28–30]. As indicated in Additional file 1: Figure S1 the analysis of chlorophyll a and b led to almost identical results between the commonly used 80% acetone method and an aliquot of 0.1 ml of the upper MTBE-fraction, indicating the suitability of this fraction for the analysis of chlorophylls. Next to the analysis of chlorophylls using the spectroscopic method, we have also validated the frequently used method of Fraser et al., for the HPLC-based analysis of carotenoids. Here we observed that the hydrophobic MTBE fraction provided comparable results for carotenoids to the results obtained using the extraction protocols described in the original paper [31] (Additional file 1: Figure S2).

**Fig. 3:**
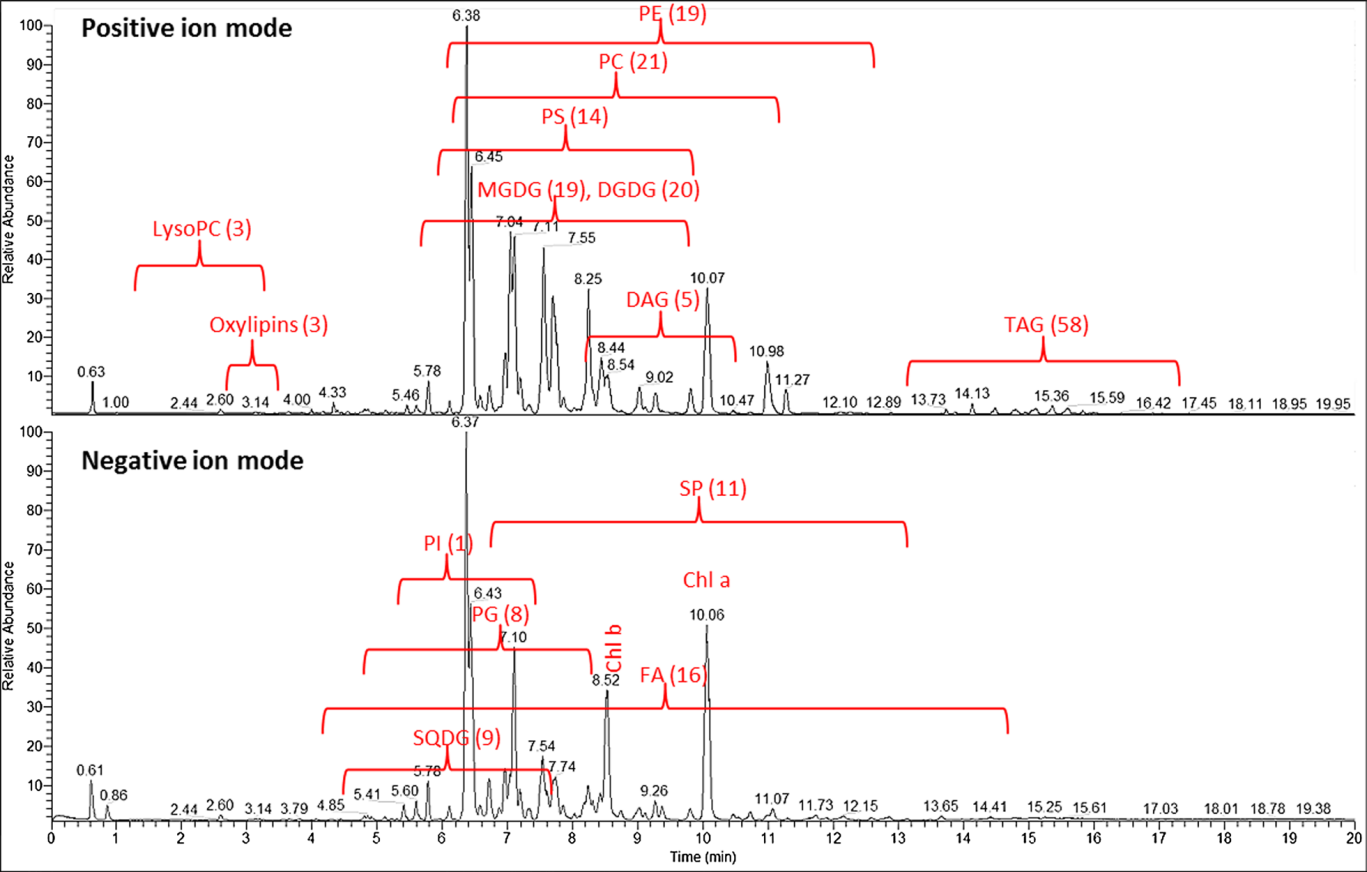
Base peak chromatograms of total lipids extracted from Arabidopsis rosette leaves. Relative abundances of eluted peaks versus retention time (min) are shown. The region of the different detected and annotated lipid classes is indicated according to its abundance either in positive or negative ion modes (for details see Additional file 1: Table S2). The number of detected lipid compounds for every class is indicated in *brackets*. *Chl a* chlorophyll a, *Chl b* chlorophyll b, *DAG* diacylglyceride, *DGDG* digalactosyldiacylglycerol, *FA* fatty acid, *LysoPC* lysophosphatidylcholine, *MGDG* monogalactosyldiacylglycerol, *PC* phosphatidylcholine, *PE* phosphatidylethanolamine, *PG* phosphatidylglycerol, *PI* phosphatidylinositol, *PS* phosphatidylserine, *SP* sphingolipid, *SQDG* sulfoquinovosyldiacylglycerol, *TAG* triacylglyceride

There are several simple (e.g. thin layer chromatography or LC-Evaporative Light Scattering detector) and advanced (UPLC-MS or Shotgun MS) methods available to analyze or profile lipids in a targeted or untargeted way [5]. One of the well-established approaches relies on the mass spectrometric analysis in combination with reversed-phase chromatographic separation [27]. This approach, especially, if fast UPLC is used, allows for the detailed profiling of the main lipid classes. The obtained data (Additional file 1: Table S2) can provide an overview of changes in the composition of the plasma-, the endogenous-, organelle- and the chloroplast membrane system, next to the availability and composition of free fatty acids and storage lipids. As described in detail in the method section, our UPLC-MS-based profiling approach enables the reliable and robust detection of more several thousand chromatographic peaks, of which at least 200 lipid species (Additional file 1: Table S2) from an Arabidopsis rosette leaf sample could be reliably annotated using the accurate mass and the obtained retention time [27]. These annotated lipid classes, obtained from the positive and negative ionization mode measurements of the same sample are displayed on a representative set of chromatograms in Fig. 3. Because each optimized chromatographic run takes only 24 min, the employed method is highly compatible to high throughput analysis of large lipidomic data sets [27, 32].

### Analysis of the polar phase: primary and secondary metabolites

As described in Fig. 2, two aliquots derived from the polar (lower) fraction, were analyzed using two complementary analytical methods. Polar primary metabolites were measured routinely, after a trimethylsilyl (TMS) derivatization, by a very well established GC–MS analysis method [21, 32], while the semi-polar secondary metabolites could be directly analyzed using a robust reversed phase UPLC-MS method [23].

**Fig. 4:**
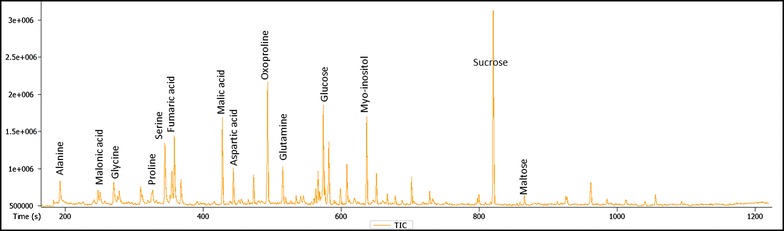
GC–MS-based total ion chromatogram of derivatized primary metabolites from Arabidopsis rosette leaves. Intensity of eluted peaks versus retention time (seconds) are shown. Identities of the most abundant metabolites are indicated. More than 90 compounds from this GC-MS data were annotated (Additional file 1: Table S3). These compounds include amino acids and their derivatives, sugars, sugar acids, sugar alcohols, sugar derivatives, organic acids and their derivatives, fatty acids, sinapates, amines and others

As can be seen from Additional file 1: Table S3, the GC–MS analysis enables the reliable detection of several hundred peaks, of which more than 90 polar metabolites, covering a wide range of metabolic classes from the central primary metabolism, including the main sugars, amino acids and organic acids, could be annotated. Figure 4 shows a representative GC–MS chromatogram, where the identities of the major metabolites are indicated.

**Fig. 5:**
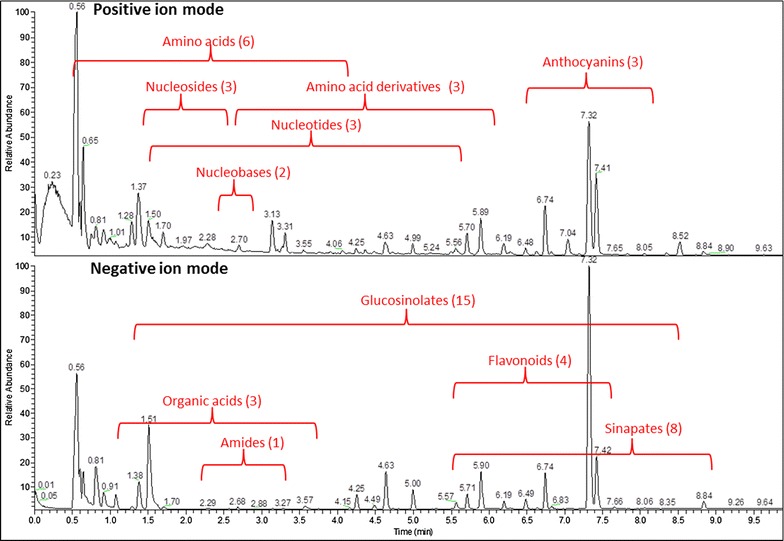
UPLC-MS base peak chromatograms of polar to semi-polar metabolites extracted from Arabidopsis rosette leaves. Relative abundances of eluted peaks versus retention time (min) are shown. The region of each eluted compound class is indicated according to its elution window either in positive or negative ionization modes. The number of detected compounds for every class is indicated in brackets (for details see Additional file 1: Table S4)

As mentioned above, next to the polar metabolites, we also annotated more than 50 secondary metabolites from rosette leaf tissue using a high-throughput UPLC-MS analysis (Additional file 1: Table S4). Similar to the lipidomic analysis, the compounds were chromatographically separated and the molecular ions were detected in the mass spectrometer using the positive and negative ionization mode (Fig. 5). In sum, these two measurements allowed us to detect, similarly to the UPLC-MS spectra from the lipidomic analysis, several thousand reproducible peaks in positive and negative ionization mode. Still, even though several thousand chromatographic peaks can be detected from this fraction, thus far only few compounds could be reliably annotated. Nevertheless, these annotated metabolites cover a wide range of the *Arabidopsis thaliana* secondary metabolism, providing a detailed insight into the regulation of the main classes of sinapates [45], glucosinolates [46], flavonoids and anthocyanins [47] (Fig. 5), which are known to be involved in many biotic and abiotic stress responses.

### Analysis of the solid pellet

**Fig. 6:**
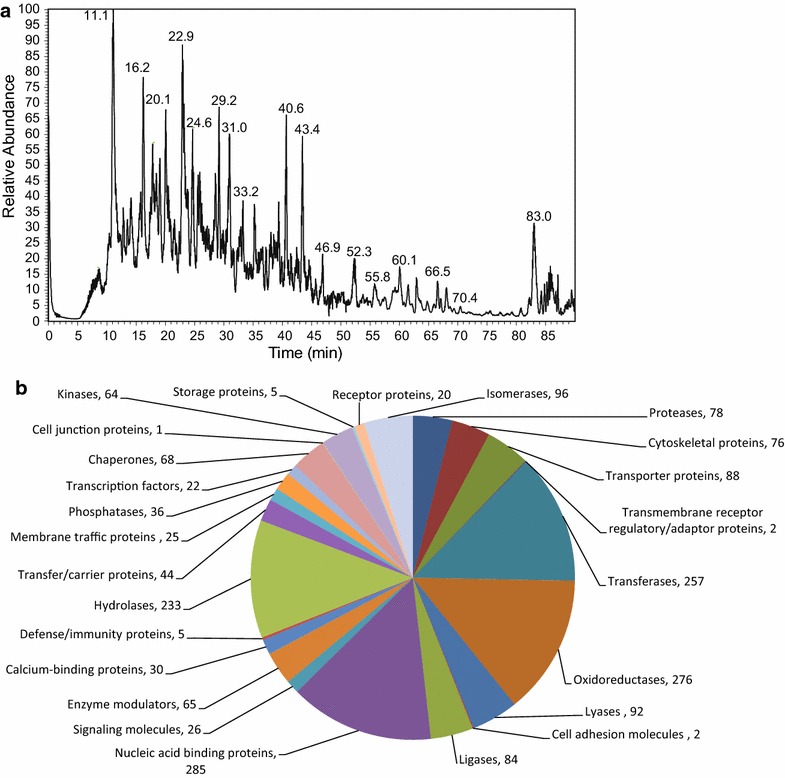
UPLC-MS chromatogram of proteins from Arabidopsis rosette leaves and their classification according to protein identifications. **a** Total ion chromatogram of proteins extracted from Arabidopsis rosette leaves. Relative abundances of eluted peaks versus retention time (min) are shown. **b** Different protein classes and the number of proteins that contribute to each class from Arabidopsis rosette leaves (for details see Additional file 1: Table S5)

After removing the liquid-extracted metabolites (polar and hydrophobic), the remaining solid pellet can be used for the extraction of proteins, starch and cell wall material (Fig. 2). The order of the extraction of the different classes of compounds cannot be interchanged, since severe losses of proteins are observed if the extraction steps required for solubilization and hydrolysis of the starch are applied before protein extraction (Additional file 1: Figure S3). Accordingly, the first step of the three-step extraction procedure of the solid pellet relies on the efficient extraction of proteins from the obtained pellet. In addition to the reproducible results of protein concentrations obtained by our extraction method (Additional file 1: Figure S3), we were able to obtain high-quality shotgun proteomics data from the generated protein extracts (Fig. 6). The results and the spectra from the proof of concept in-solution digestion and nanoLC-MS analysis of the extracted proteins allowed for the routine identification of more than 2000 proteins from a 25 mg sample of *Arabidopsis thaliana* rosette leaves. The obtained identifications, using a single measurement, had at least two independent peptides and a false discovery rate (FDR) below 1% (Additional file 1: Table S5). Interestingly, next to the large amount of soluble proteins, we also detected significant amounts of transmembrane proteins, especially from the thylakoids, the nucleus, the ER, and the plasma membrane. This increased quantity of hydrophobic proteins is explained by the fact that the MTBE extraction method provides a clean and completely de-lipidation of membranes, namely membrane lipid are extracted in the upper MTBE phase, providing a high quantity of precipitated membrane proteins in the solid pellet.

Once the proteins are extracted from the solid pellet, a quantitative extraction and analysis of cellular starch content can be performed on the remaining pellet. Since starch analysis is a highly established and standardized method for photoautotrophic organisms, we aimed to compare the extracted amounts of starch derived from the insoluble protein, starch, cell wall pellet obtained from the MTBE extraction, to the values obtained from the commonly used standard extraction methods [32, 36]. Much as we hoped for, we found that the starch concentrations obtained from the de-proteinated pellet were highly similar to the concentrations measured by the Smith and Zeeman protocol [36]. Next to the loss-free fractionated extraction of protein and starch, our method also proved to be highly reproducible over a large range of concentrations as indicated by the small error bars (Additional file 1: Figure S4).

Once we reached the de-proteinated and de-starched pellet at the end of the sequential extraction of the solid pellet, there is still material for an additional analysis left, namely the insoluble cell wall material. This material can be used to determine the polysaccharide composition of cell walls, the crystalline cellulose content and lignin by GC–MS and spectroscopic methods (see “Methods” section). Additional file 1: Figure S5 summarizes the analysis of this last fraction of the solid pellet and illustrated that these compounds can be analyzed reproducibly from the remaining pellet, providing an additional insight in an often-neglected cellular compartment.

## Discussion

The application of the here described protocol, allows for the independent and qualitative extraction and separation of the major compound classes, from a single sample. In addition to the detailed extraction protocol (see “Methods” section), we additionally provide exemplary analytical data, mainly using gas- (GC) and liquid-chromatography (LC) coupled to diverse mass spectrometers for the analysis of the three different phases (organic, polar and solid). The precise conditions for analysis of different metabolites are based on the availability of specific instruments and can be easily extended beyond the provided examples given in this article.

As we have shown in this protocol, the analysis of a single sample using the fractionated extraction method, provides profound insight, not only into diverse molecular compounds, but also provides a functional overview of most cellular compartments and processes. The combination of these divers molecular entities, especially the combination of metabolite data and the protein data allows to draw causal conclusions of the functional molecular machines (proteins) and their products (metabolites), from the exact same sample. The use of a single sample therefore allows minimizing the difference between the origin of measurement and therefore to maximize the accuracy of the analysis. Next to the decrease in sample consumption, this strategy provides an ideal foundation for computational systems biological approaches.

### Applications of the MTBE extraction protocol for ‘omic’-based analysis

As described in the result section the total amount of 25 mg of leaf tissue allows for the complex analysis of several hundred molecular properties of a single sample. Of course, the analysis and the annotation of further compounds are only limited by the biological question and the analytical methods and the equipment employed for the downstream analysis of the obtained extracts. Thus far we have not encountered analytical methods that were incompatible with the obtained fractions and in most cases the obtained abundance and the quality of the compounds from the MTBE-derived extracts was reaching sensitivities and quality to the more specialized extraction methods. Beyond the optimal applicability of our method for plant cells and tissues, it should be mentioned that we have not only applied this method thus far for diverse plant samples [23, 27, 32, 48–54], but it was also successfully employed for metabolic and/or proteomic studies of algae [55, 56], flies [57] and diverse mammalian cells and tissues [58–60].

In previous studies, we have analyzed the MTBE-derived lipid phase for the identification and analysis of lipid species from *Arabidopsis thaliana* dry seeds [48, 53], seedlings [32, 49], leaves [23, 27, 50–54], roots [23] and flowers [54]. Additionally, we applied the same method, with minor adaptations in the extraction process, to compare the lipid composition of mammalian tissues including brain, kidney and skeletal muscle of mice, rhesus macaques, chimpanzees and humans [58–60]. Moreover, the method allowed the detection and annotation of more than 180 lipid species from *Chlamydomonas reinhardtii* [55]. Furthermore, we applied the described method to determine the lipid composition of the green algal species *Scenedesmus (Acutodesmus) obliquus* [56] and the model fly *Drosophila melanogaster* [57]. Moreover, the method has been proven useful also for lipid profiling of 124 lipid species from the marine diatom (*Thalassiosira pseudonana*) [53, 61] and the biddulphioid diatom (*Biddulphia biddulphiana*) [61].

Next to the major lipid profiling approaches described above, we also applied the extraction method for the analysis of polar and semi-polar compounds in several plant species. These analyses provided a basic insight into central carbon and nitrogen metabolism at the systems level. Accordingly, we were able to apply the protocol for the extraction, detection and identification of primary and/or secondary metabolites of several species and tissues. Amongst others, we studied *Arabidopsis thaliana* seedlings [32], roots and leaves [23], barley (*Hordeum vulgare*) [62], wild strawberry (*Fragaria vesca*) [63] strawberry (*Fragaria* X *ananassa*) [64], the root tissue of maize (*Zea mays*) [65], the green algae *Chlamydomonas reinhardtii* [66], the marine diatom *Thalassiosira pseudonana* [61] and some low phosphate-tolerant proteaceae species [51].

### Future perspectives and challenges

In this protocol, we showed that our method could be used for a comprehensive “multi-omics” sample extraction, preparation and analysis. The analysis of multiple molecular entities, derived from several subcellular compartments and molecular processes provides a brought overview of the status of the cell. Still compound annotation and/or identification are the major challenge in the metabolomics data analysis. Although we were able to annotate many lipids, metabolites and proteins, more compounds are still to be uncovered and would, if possible allow to broaden our insight into the molecular inventory of the cell. For instance, even though we detected some sphingolipids or sterols in our lipid analysis, these lipid specific lipid classes are slightly underrepresented in our data set. This underrepresentation is not due to the extraction procedure, but it is explained by the complexity of the sample, namely by ion suppression or matrix effects, and the measurement mode of our method. Still, it should be easily possible to modify our analytical workflow enabling the inclusion of the missed compounds. For instance, it is possible to expand the number of detectable sphingolipids by analyzing the MTBE but also the methanol phase after mild base hydrolysis, which allows depleting the highly abundant glycerolipids and therefore improve the analysis of the slightly less abundant 100–150 species of the non-hydrolysable sphingolipids [67, 68]. Next to the dedicated analysis of sphingolipids, the uses of either atmospheric pressure chemical ionization (APCI) or improved direct infusion MS-based analysis strategies can be used for the analysis of more than 100 sterols and their derivatives [69, 70].

As already mentioned in the result section, we do routinely annotate 50–60 secondary metabolites from *Arabidopsis thaliana* by our UPLC–MS analysis. Although these secondary metabolite classes include the main secondary metabolites like the sinapates, glucosinolates, flavonoids and anthocyanins, still we have to admit that the obtained spectra from these UPLC-MS measurements contain plenty of reproducible but unidentified chromatographic peaks. Many of these will be true secondary metabolites derived from the plant. In a previous paper using the polar phase from the MTBE extraction method, we demonstrated by multiple isotope labelling experiments that more than 1400 chromatographic peaks of these spectra, obtained from the UPLC-MS analysis of the polar fraction, were of biological origin, indicating the large pool of biologically-relevant information contained and unexploited in these samples [23]. Unfortunately, metabolite annotation is still far from routine and high-throughput [71]. The main complication by using this fraction for the analysis of secondary metabolites comes more from the high structural complexity of the analyzed compounds and the difficulty to annotate them without authentic reference compounds. In the above-mentioned study, we were using stable isotope labeling for the unambiguous annotation of the compounds, but of course it would be desirable to additionally use authentic standards and higher order MS-based fragmentation analysis for the proper structural elucidation of the thus far unknown compounds.

Regarding proteomic analysis, we routinely identify 2000–3000 proteins from Arabidopsis covering several enzymes, signaling- and transmembrane proteins. The number of specific proteins can still be increased by either increasing the amount of starting material but also by sub fractionation of the obtained pellet. In the current protocol, we re-suspended the protein pellet for example initially in urea/thiourea buffer, which might not fully solubilize the most hydrophobic membrane proteins. Accordingly the protein extraction buffer, could be changed to a detergent-containing buffer (e.g. a 0.5–1% sodium dodecyl sulphate-containg buffer) in combination with a Filter Aided Sample Preparation (FASP) in solution digestion protocol [72].

It is also possible to maximize the number of extractable and identifiable proteins by using a sequential protein extraction strategy [73]. So one can start extracting the soluble proteins with a mild Tris buffered saline (TBS) buffer, followed by the extraction of structure associated proteins using a stronger chaotropic buffer (e.g. urea/thio urea) and finally, as mentioned above, a detergent-containing buffer for the extraction of transmembrane proteins. Still, it has to be taken into account that every additional fractionation maximizes the sample number and therefore the analysis effort and time.

Next to the sequential analysis of specific proteins, one can also envision to use the obtained protein extracts for the analysis of specifically modified proteins. For this purpose multiple enrichment strategies for e.g. phosphorylated or glycosylated proteins or peptides are readily available [74, 75]. Not to mention, that the obtained protein extract can be efficiently used for gel-based separation in combination with mass spectrometry-based proteomic analysis, 2D-gel-based proteomics, phosphoproteomics or simply western blotting [73, 76].

## Conclusion

In this protocol, we describe a universal extraction method that allows for the preparation and isolation of lipids, metabolites, proteins and other macromolecules for high-throughput multi-omics analysis using a single biological plant sample. We optimized and used this approach to generate several analytical datasets from the same sample. This allows for the brought insight into the analyzed system and decreases the bias in systems biology application. Accordingly, the described method does not only lead to significantly reduced sample consumption but also minimizes the time and effort needed to perform separate extractions when many molecules are to be studied in the same experiment. On the long run, we are planning to further improve the method, especially by expanding the repertoire of applicable analytical methods and therefore further broadening the number and quality of the detectable components.

## Additional file

**Additional file 1.** Supplemental Figures and Tables.
