# Supplementary material for: Protocol: a fast, comprehensive and reproducible one-step extraction method for the rapid preparation of polar and semi-polar metabolites, lipids, proteins, starch and cell wall polymers from a single sample
Source: Plant Methods. 2016 Nov 10;12:45. doi: 10.1186/s13007-016-0146-2 (PMC5103428; doi:10.1186/s13007-016-0146-2)
Supplement: Supplementary file 1 — Additional file 1. Supplemental Figures and Tables. [file 13007_2016_146_MOESM1_ESM.zip › Additional file.docx]

**Additional file 1**

**Supplemental tables**

**Table S1: Instructions details for efficient extraction method.**

**Table S2:** **Result table of the annotated lipids measured by UPLC-MS**

**Table S3: Result table of the annotated metabolites measured by GC-MS**

**Table S4: Result table of the annotated metabolites measured by UPLC-MS**

**Table S5: Result table of the identified proteins measured by nLC-MS**

**Supplemental figures**

**Figure S1. Chlorophyll content of young wild-type Arabidopsis leaves following MTBE-based and a reference extraction protocol.**

Chlorophyll was extracted from 21-day-old wild-type *Arabidopsis thaliana* plants grown under long day conditions. Chlorophyll was extracted following the MTBE protocol (see Methods) and Ni et al. 2009. Error bars indicate +/- SD for at least 5 biological replicates from at least 2 independent experiments.


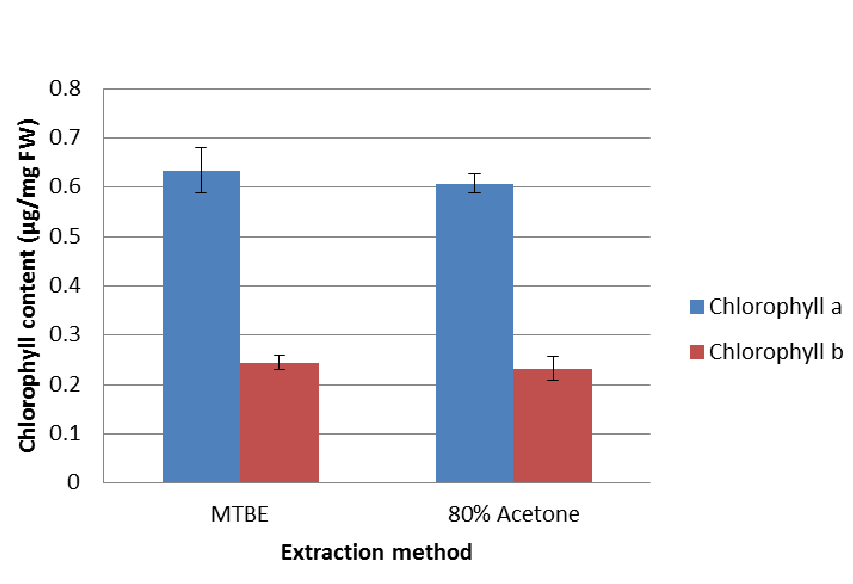


**Figure S2. Pigment content in wild-type Arabidopsis leaves following MTBE-based and a reference extraction protocol.**

Pigments were extracted from 30-day-old wild-type *Arabidopsis thaliana* plants grown under long day conditions. Pigments were extracted following the MTBE protocol (see Methods) and Fraser et al. (2007). Pigments were analyzed by high-performance liquid chromatography (HPLC) with photodiode array (PDA) detection following Fraser et al. (2007). Error bars indicate +/- SD for at least 5 biological replicates from at least 2 independent experiments.

**Figure S3. Influence of order of protein and starch extraction on extractable contents.**

Protein and starch were extracted from 30-day-old wild-type *Arabidopsis thaliana* plants grown under long day conditions following this protocol (see Methods). Error bars indicate +/- SD for at least 5 biological replicates from at least 2 independent experiments. *significantly different from the wild-type under the same condition (P<0.001, Student’s *t*-test).

**Figure S4. Starch content in wild-type Arabidopsis leaves following MTBE-based and a reference extraction protocol.**

Starch was extracted from 30-day-old wild-type *Arabidopsis thaliana* leaves plants grown under long day conditions. Starch was extracted following this protocol (see Methods) and Smith and Zeeman protocol (2006). Starch content was quantified in both cases following Smith and Zeeman protocol (2006). Error bars indicate +/- SD for at least 5 biological replicates from at least 2 independent experiments.

**Figure S5. Cell wall analyses of Arabidopsis leaves following the MTBE-extraction protocol**

GC-MS analysis of the monosaccharide composition of TFA hydrolysates of leaves. The cell wall material was measured from cell wall remained after MTBE extraction according to Foster et al. (2010). Sugars are presented as signal intensity (peak area x10^6^). Lignin content (mg/g FW) was measures by thioglycolic acid (TGA) method Bruce and West (1989). Crystalline cellulose content (µmol/mg CW) was determined by the Updegraff method (1969). Error bars indicate +/- SD for at least 5 biological replicates from at least 2 independent experiments.

**Supplemental references**

**Bruce RJ, West CA.** 1989. Elicitation of lignin biosynthesis and isoperoxidase activity by pectic fragments in suspension cultures of castor bean. Plant Physiology 91, 889-897.

**Foster CE, Martin TM, Pauly M.** 2010. Comprehensive compositional analysis of plant cell walls (lignocellulosic biomass) part II: carbohydrates. J Vis Exp.

**Fraser PD, Enfissi EMA, Goodfellow M, Eguchi T, Bramley PM.** 2007. Metabolite profiling of plant carotenoids using the matrix-assisted laser desorption ionization time-of-flight mass spectrometry. Plant Journal 49, 552-564.

**Ni ZF, Kim ED, Ha MS, Lackey E, Liu JX, Zhang YR, Sun QX, Chen ZJ. 2009.** Altered circadian rhythms regulate growth vigour in hybrids and allopolyploids. Nature 457, 327-U327.

**Smith AM, Zeeman SC.** 2006. Quantification of starch in plant tissues. Nat Protoc 1, 1342-1345.

**Updegraff DM.** 1969. Semimicro determination of cellulose in biological materials. Anal Biochem 32, 420-424.
