# Supplementary material for: Protocol: a fast, comprehensive and reproducible one-step extraction method for the rapid preparation of polar and semi-polar metabolites, lipids, proteins, starch and cell wall polymers from a single sample
Source: Plant Methods. 2016 Nov 10;12:45. doi: 10.1186/s13007-016-0146-2 (PMC5103428; doi:10.1186/s13007-016-0146-2)
Supplement: Supplementary file 1 — Additional file 1. Supplemental Figures and Tables. [file 13007_2016_146_MOESM1_ESM.zip › TableS1_Troubleshooting.docx]

**Table S1: Instructions details for efficient extraction method**

| **Step** | **Instructions for users** |
| --- | --- |
| Plant material harvesting | - Plant material has to be harvested and snap frozen in liquid nitrogen immediately to minimize induced changes at the molecular levels. |
| Storage of harvested tissues | - The harvested samples can be stored in liquid nitrogen or on dry ice for short-term storage, while longer-term storage should be at – 80 ºC. - Usually intact, non-ground plant material, can be stored for 1 year or more at -80 °C, while homogenized material might be more problematic, which should be lyophilized and aliquoted prior to longer term storage. |
| Grinding of plant material | - For highest extraction efficiencies, the material should be ground into a homogeneous and fine powder. Therefore either a homogenizer or if not available a classical mortar and pestle can be used. - Here it is evident that the plant material has to be kept frozen at every step of the homogenization. - If a mortar and a pestle were used for the homogenization, pre-cooling in liquid nitrogen for some minutes before starting is required. The same precautions have of course to be taken if tissue homogenizers are used. |
| Weighing of plant material | - After disintegration of the plant material, the required amount of powdered material of each sample (10-100 mg, depending on the tissue or analysis purpose) should be aliquoted into pre-labelled 2 ml safe-lock microcentrifuge tubes or glass vials with Teflon-lined scree caps. - The exact weight for each sample should be recorded, since it is required for the normalization of signal intensities. A maximal variation of ± 10% between samples should be achieved. |
| Reagent setup | - The extracting solution M1 can be stored for up to 1 week at 4 ºC or for ~1 month at – 20 ºC. We recommend to always prepare M1, including the required internal standards, freshly and in quantities needed for the actually planned experiment (1 ml / sample). This precaution allows maintaining high reproducibility between the different samples from the same experiment. - The solvent M2 induces the phase separation and can be stored for up to ~3 months at room temperature, but we recommend storage at 4 °C. - To avoid chemical noise or contaminations during analysis, highest-quality grade solvents are recommended. - It is highly advised to perform the lipid extraction steps using pre-cleaned glass equipment (vials and pipette tips) in combination with Teflon-lined screw caps (we rinse every vial and bottle with water, methanol and internal standard-free M1 solution), to avoid contamination of polypropylene or other polymers and plasticizers. Unfortunately, the use of glassware precludes the efficient use of standard tabletop centrifuges, which makes the extraction process more tedious and decreases the speed and the throughput. |
| First step of extraction | - In our standard protocol, a fixed volume (1 ml) of pre-cooled (-20°C) extraction solvent M1 was added to each tube/vial. Of course, the extraction volume can be, as well as the employed sample amounts, freely scaled. We highly recommend, if new tissue or species are to be analyzed, to run test samples, to determine the ideal volume and sample amounts for the extraction. For most of the thus far analyzed samples, it worked perfectly fine to use a volume of 1 mL in combination with 10-50 mg of tissue (fresh weight). - The pipetting of M1 has to be performed quickly but also carefully, since MTBE has a very low viscosity and tends to drip out of the pipette tips, which might introduce volume errors. - The frozen and powdered samples should be stored in a rack on dry ice or liquid nitrogen to avoid any sample thawing before complete mixing of the powder with denaturing solvent M1. - The mixing step has to be performed quickly to make sure that the entire material is re-suspended, proteins are immediately denatured and undesired enzyme activities are minimized. - The sonication supports the disruption of large particulate plant material and improves the solubilization of the contained metabolites. For pre-cooling of the sonication bath, we add a handful of ice to the water. |
| Two phase separation | - Centrifugation leads to a clear separation of the two liquid phases and the pelleting of the insoluble material in the bottom of the vial/tube. The upper phase (usually green, if green tissue was used) contains polar- and neutral lipids and non-polar pigments, while the lower phase (methanol/water) contains the polar to semi-polar metabolites like e.g. amino acids and sugars. The pellet, which should be white at this point, even if green tissue was used, contains proteins, starch and insoluble cell wall material. - After the phase separation step, the tubes have to be handled carefully to avoid mixing of the two liquid phases and to avoid disrupting the precipitated pellet. - If larger amounts of leaf tissue (more than 50 mg of *A. thaliana*) or high starch-containing plants (harvested at the end of the day) were used for the extraction, a solid interphase between the two liquid phases can be obtained. This interphase usually contained almost only starch and can be avoided by reducing the sample amounts of the starting material. In our experiments, we never need to use more the 25-50 mg (fresh weight) of tissue powder. If large quantities of starch-containing tissues are required and an interphase is formed, we suggest to collect the complete organic, lipid-containing, phase and add an additional 150 µl of extraction solvent M2 to each vial/tube. After a short vortexing step, the sample is centrifuged at a speed of 20,000*g* for another 10 min at 4 ºC. Usually the interphase will be completely pelleted with the other insoluble material. One precaution has to be still taken, since the total volume of the polar phase was increased. The added volume has to be taken into account in the collection of aliquots for the downstream analysis to allow proper quantification and inter-experimental comparisons. |
| Analysis of lipids | - For the evaporation, we use either a SpeedVac concentrator at RT or, preferably, a nitrogen flow evaporator, which helps minimizing sample oxidation. - If samples are supposed to be analyzed later, we recommend to store the samples at -80 °C in solution (MTBE phase) rather than storing the dried pellet, which minimizes oxidative degradation. - The re-suspension volume is depending on the type and amount of the employed tissue used for the extraction. Seeds, which are lipid-rich, might require stronger dilution than root tissue, which usually containing less lipids. We highly recommend, if new tissue or species are to be analyzed, to run test samples, to determine the ideal volume of the re-suspension solution. Keep in mind: it is easier to further dilute than to concentrate. - If the chlorophyll and carotenoid contents are to be determined, the whole extraction and pre-measurement treatment should ideally be performed in the dark, since pigments are sensitive to light. |
| Analysis of polar metabolites | - The dried samples from the polar phase can either be directly analyzed (preferential) or stored at -80 ºC for up a year until analysis. Flushing the tubes with argon or nitrogen before long-term storage, will help to prevent oxidative damage during storage. Air, water and light should be avoided during storage. - The re-suspension volume is depending on the type and amount of the employed tissue used for the extraction. We recommend, if new tissue or species are to be analyzed, to run test samples, to determine the ideal volume of the re-suspension solution. |
| Protein/ starch extraction | - If both starch and proteins are supposed to be analyzed from the same sample, the order of extraction is extremely important. Here it is extremely relevant that the proteins have to be extracted before starch, since the heating steps during starch extraction leads to severe protein losses, while the pre-extraction of proteins had no impact on detectable starch quantities. In this step, it is important not to remove any part of the precipitated pellet. - The washed pellets can then either be kept on ice and directly processed or stored at – 80 ºC until further extraction. We recommend to perform the starch and/or proteins analysis directly and avoid long term storage to prevent degradations of the samples. - The re-suspension volume has of course to be adjusted to the employed type and amount of sample. We recommend the use of 200 µl protein extraction buffer for 50 mg plant material. - Heating of the extraction solvent containing urea should be avoided because this can cause decomposition and protein modification with cyanate. |
